# Localization with a Mobile Beacon in Underwater Acoustic Sensor Networks

**DOI:** 10.3390/s120505486

**Published:** 2012-04-27

**Authors:** Sangho Lee, Kiseon Kim

**Affiliations:** School of Information and Mechatronics, Department of Nanobio Materials and Electronics, WCU, Gwangju Institute of Science and Technology (GIST), 1 Oryong-dong, Buk-Gu, Gwangju 500-712, Korea; E-Mail: faith.kskim@gist.ac.kr

**Keywords:** localization, range-free, mobile beacon, underwater acoustic sensor networks

## Abstract

Localization is one of the most important issues associated with underwater acoustic sensor networks, especially when sensor nodes are randomly deployed. Given that it is difficult to deploy beacon nodes at predetermined locations, localization schemes with a mobile beacon on the sea surface or along the planned path are inherently convenient, accurate, and energy-efficient. In this paper, we propose a new range-free Localization with a Mobile Beacon (LoMoB). The mobile beacon periodically broadcasts a beacon message containing its location. Sensor nodes are individually localized by passively receiving the beacon messages without inter-node communications. For location estimation, a set of potential locations are obtained as candidates for a node's location and then the node's location is determined through the weighted mean of all the potential locations with the weights computed based on residuals.

## 1. Introduction

For a long time, there has been significant interest in monitoring underwater environments to collect oceanographic data and to explore underwater resources. These harsh underwater environments have limited human access and most of them remain poorly understood. Recent advances in hardware and network technology had enabled sensor networks capable of sensing, data processing, and communication. A collection of sensor nodes, which have a limited sensing region, processing power, and energy, can be randomly deployed and connected to form a network in order to monitor a wide area. Sensor networks are particularly promising for use in underwater environments where access is difficult [1–3]. In underwater acoustic sensor networks (UASN), localization of sensor nodes is essential because it is difficult to accurately deploy sensor nodes to predetermined locations. In addition, all the information collected by sensor nodes may be useless unless the location of each sensor node is known [4].

**Figure 1.**
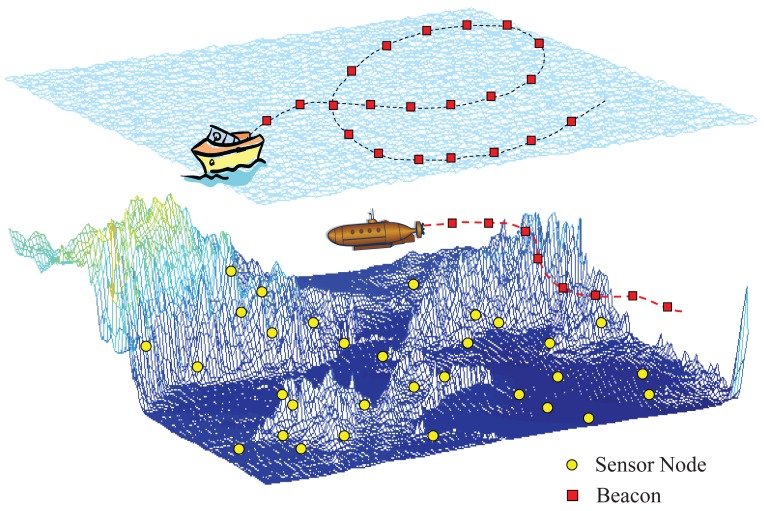
Movement of a mobile beacon for localization in underwater environments.

To localize unknown nodes, static beacons [5–11] or a mobile beacon [12–16] can be used; typically, buoys are used for static beacons and an autonomous underwater vehicle (AUV) for a mobile beacon. The use of a mobile beacon has a similar effect to the use of many static beacons. For this reason, localization using a mobile beacon is inherently more accurate and cost-effective than localization using static beacons. Further, the vehicle can be conveniently maneuvered on the sea surface or underwater, based on a planned or random path, as shown in Figure 1.

Localization schemes can also be classified as range-based or range-free schemes. Range-based schemes use the distance and/or angle information measured by the Time of Arrival (ToA), Time Difference of Arrival (TDoA), Angle of Arrival (AoA), or Received Signal Strength Indicator (RSSI) techniques. In underwater environments, range-based schemes that use the ToA and TDoA techniques have been proposed [5–10,13–15]. Time synchronization between sensor nodes is typically required for the ToA and TDoA techniques [6]. However, precise time synchronization is challenging in underwater environments [17]. In addition, even though the ToA and TDoA techniques are used without time synchronization, the techniques are based on the speed of sound and this varies with temperature, pressure, salinity, and depth. In contrast to range-based schemes, range-free schemes localize a sensor node by deducing distance information instead of measuring distances [11,12,16]. Because range-free schemes do not require additional devices in order to measure distances and do not suffer from distance measurement errors, range-free schemes are a promising approach for underwater sensor networks [16].

Localization is one of the challenges associated with underwater sensor networks and many studies have focused on localization in recent years. Among these studies, LDB is a range-free localization scheme with a mobile beacon [16]. In LDB, sensor nodes can localize themselves through passively listening to beacons sent by an AUV that has an acoustic directional transceiver. Because sensor nodes just receive the beacons from the AUV without inter-node communications, LDB is energy-efficient because it reduces the energy consumption due to transmitting; the power consumed by transmitting is often 100 times higher than the power consumed by receiving [18]. In addition, because a sensor node can localize itself using its own received beacons from the AUV independently of the other sensor nodes, LDB has fine-grained accuracy even in sparse networks. The depth of a sensor node is directly determined by using a cheap pressure sensor. After the first and last received beacons, which are called beacon points, from the AUV are projected onto the horizontal plane on which a sensor node resides, the 2D location of a sensor node can be determined based on the distance between the two projected beacon points. At this point, the location of the sensor node remains ambiguous, because the two projected beacon points provide two possible node locations. If the sensor node obtains two more beacon points, the ambiguity can be resolved. The first two beacon points are used for location computation and the other two beacon points are used for resolving the ambiguity. However, if the two beacon points for location computation have a large error, the error in the estimated sensor location also increases. Even though the location of a sensor node can be simply computed from the first two beacon points, the use of just two points out of all the beacon points can make localization very error-prone. To improve the localization accuracy in challenging underwater environments, we propose a new localization scheme LoMoB that obtains a set of potential locations as candidates for a node's location using the bilateration method and then localizes the sensor node through the weighted mean of all the potential locations with the weights computed based on residuals.

## 2. Related Work

In this section, we briefly explain LDB in the viewpoint of system environment, beacon point selection, and location estimation [16].

### 2.1. System Environment

**Figure 2.**
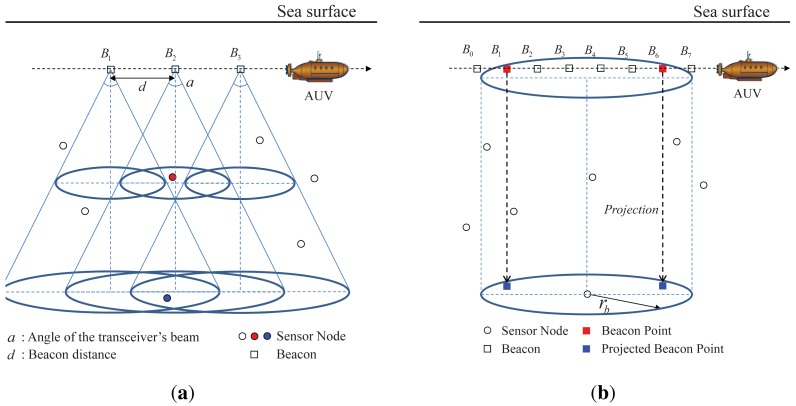
System environment and beacon point selection and projection for LDB. (**a**) System environment with an AUV that has a directional transceiver; (**b**) Selection and projection of beacon points from among the received beacons.

In LDB, an AUV with a directional transceiver moves at a fixed depth in water and broadcasts its location, which called a beacon, and the angle of its transceiver's beam (*a*) at regular intervals, called beacon distance (*d*), as shown in Figure 2(a). When the AUV sends a beacon, the sensor nodes that fall in the conical beam receive the beacon; e.g., in Figure 2(a), the sensor node in red can receive the beacon *B*_2_ and the sensor node in blue can receive the beacons *B*_1_, *B*_2_, and *B*_3_. The conical beam forms circles with different radii according to the depth of the sensor nodes, as shown in Figure 2(a). When the *z* coordinates of the AUV and a sensor node are *z_A_* and *z_s_* and the angle of the transceiver's beam is *a,* the radius of the circle formed by the beam for the sensor node is given as follows:

(1) $$r_{b}=\tan(a/2)\times \left|z_{A}-z_{s}\right|$$

### 2.2. Beacon Point Selection

From the viewpoint of a sensor node located at (*x, y, z_s_*), when a beacon is within the circle centered at (*x, y, z_A_*) with a radius *r_b_*, the sensor node can receive the beacon; e.g., in Figure 2(b), the sensor node receives the beacons *B*_1_ to *B*_6_. Among the series of received beacons, the first beacon is defined as the first-heard beacon point and the last beacon is defined as the last-heard beacon point. The location of a sensor node is estimated using only the beacon points, and not all the received beacons.

Because the sensor depth is known from the pressure sensor, the beacon points can be projected onto the horizontal plane in which the sensor node resides, as shown in Figure 2(b). After projection, 3D localization is transformed into a 2D localization problem.

### 2.3. Location Estimation

**Figure 3.**
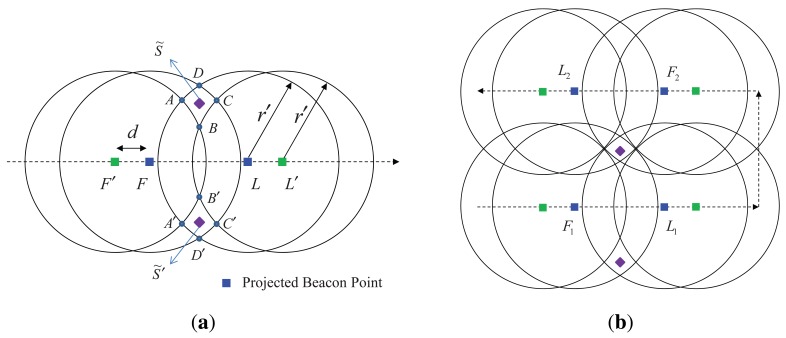
Location estimation with projected beacon points based on geometric constraints. (**a**) Location estimation based on two projected beacon points; (**b**) Location decision using two additional projected beacon points.

We describe location estimation based on the projected beacon points. As shown in Figure 3(a), the projected first-heard beacon point is denoted by *F* and the projected last-heard beacon point is denoted by *L.* The projected point of the beacon received just before the first-heard beacon point is defined as the projected prior-heard beacon point *F*′ (e.g., in Figure 2(b), the projected point of *B*_0_), and the projected point of the beacon after the last-heard beacon point is defined as the projected post-heard beacon point *L*′ (e.g., in Figure 2(b), the projected point of *B*_7_).

Four circles are then drawn with radius *r_b_* centered at the four points *F*′, *F, L*, and *L*′. Because the sensor node should be located outside the circles centered at *F*′ and *L*′ and inside the circles centered at *F* and *L*, the intersection areas *ABCD* and *A*′*B*′*C*′*D*′ represent possible locations of the sensor node. One of the two points, *S̃* and *S̃*′, within the two areas will be estimated as the location of the sensor node. Here, *S̃* is the midpoint of *B* and *D* and *S̃*′ is the midpoint of *B*′ and *D*′. When just one projected first-heard beacon point and one projected last-heard beacon point are used, two possible node locations are found. The choice between the two points *S̃* and *S̃*′ can be made after an additional two beacon points are obtained, as shown in Figure 3(b). In LDB, the first two beacon points are used to compute two possible points for the location of a sensor node based on geometric constraints and the next two beacon points are used for the choice between the two possible points.

## 3. LoMoB Localization Scheme

In this section, we explain the system environment, beacon point selection, and location estimation method for LoMoB. Because LoMoB can be applied to systems that use either a directional transceiver or an omnidirectional transceiver, we explain LoMoB for both systems. In addition, because LoMoB improves LDB, we explain LoMoB by comparing and contrasting it with LDB.

### 3.1. System Environment

LoMoB considers a system that uses an omnidirectional transceiver in addition to a system that uses a directional transceiver [19]. Because the system environment using a directional transceiver was explained in Section 2, we explain here the system environment using an omnidirectional transceiver.

**Figure 4.**
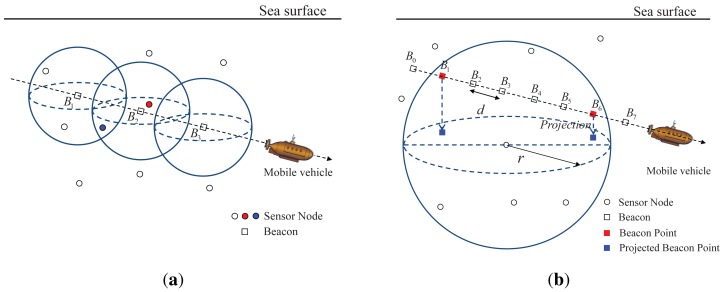
System environment, beacon point selection, and projection for LoMoB. (**a**) System environment with a mobile beacon that has an omnidirectional transceiver; (**b**) Selection and projection of beacon points from among the received beacons.

A mobile beacon moves on the sea surface or underwater. When a mobile beacon has an omnidirectional transceiver, 3D movement of the mobile beacon is possible whereas a mobile beacon that has a directional transceiver is restricted to 2D movement at a fixed depth. The mobile beacon is assumed to know its own location and to broadcast a beacon containing its location information at regular distance intervals, which are called the beacon distance *d*. When the mobile beacon transmits a beacon, the sensor nodes that are located within the communication range of the mobile beacon receive the beacon; e.g., in Figure 4(a), the sensor node in red can receive the beacon *B*_2_ and the sensor node in blue can receive the beacons *B*_1_ and *B*_2_. Here, the communication range *r* is assumed to be constant in 3D [11,12,20]. In addition, because movement of a mobile beacon in a straight line is more controllable than curved movement, the mobile beacon is assumed to follow the random waypoint (RWP) model [21]; the mobile beacon moves in a series of straight paths to random destinations.

### 3.2. Beacon Point Selection

From the viewpoint of a sensor node, the sensor node can receive a beacon when the beacon is within the communication range of the sensor node; e.g., in Figure 4(b), the sensor node receives the beacons *B*_1_ to *B*_6_. The selection of beacon points from the received beacons is performed as in LDB. When a sensor node receives the first beacon from a mobile beacon, the first beacon is selected as a beacon point; *i.e., B*_1_ in Figure 4(b). If the sensor node receives no further beacons during a predefined time after receiving its last beacon, the last beacon is selected as a beacon point; *i.e., B*_6_ in Figure 4(b). The above process is repeated each time the mobile beacon passes through the communication sphere of the sensor node.

As shown in Figure 4(b), the beacon points do not lie exactly on the communication sphere of the sensor node, because the sensor node receives a beacon at every beacon distance *d*. When the communication range of the sensor node is *r*, the beacon points are located between the distances *r* − *d* and *r* from the sensor node. Based on the distance range between a beacon point and a sensor node, the middle value of the range, *r* − *d*/2, is estimated as the distance between the beacon point and sensor node in order to minimize the error in the estimated distance.

Sensor nodes are assumed to have a pressure sensor, and therefore they are assumed to know their depth [22]. With this depth information, the beacon points can be projected onto the horizontal plane in which the sensor node resides, as shown in Figure 4(b). When the *z* coordinates of a sensor node and the *i^th^* beacon point are *z_s_* and *z_i_*, the distance $r_{i}^{\prime }$ between the sensor node and the *i^th^* projected beacon point is $\sqrt{{\left(r-d/2\right)}^{2}-{\left(z_{i}-z_{s}\right)}^{2}}$.

For a system using a directional transceiver, all the projected beacon points are located between the distances *r_b_* − *d* and *r_b_* from a sensor node; here, *r_b_* is the radius of the circle formed by the beam for the sensor node. Subsequently, all the estimated distances between the sensor node and projected beacon points are identically *r_b_* − *d*/2.

### 3.3. Location Estimation

In this subsection, we explain how to estimate the location of a sensor node based on the distances between the sensor node and the projected beacon points. First, potential locations are obtained as candidates for the location of the sensor node. Second, the location of the sensor node is estimated using the weighted mean of the potential locations.

#### 3.3.1. Obtaining potential locations

After estimating the distances between a sensor node and projected beacon points, the following equations need to be solved for localization:

(2) $$\{\begin{array}{l} \sqrt{{\left(x-x_{1}^{\prime }\right)}^{2}+{\left(y-y_{1}^{\prime }\right)}^{2}}=r_{1}^{\prime }\\ \sqrt{{\left(x-x_{2}^{\prime }\right)}^{2}+{\left(y-y_{2}^{\prime }\right)}^{2}}=r_{2}^{\prime }\\ \;\vdots \\ \sqrt{{\left(x-x_{N}^{\prime }\right)}^{2}+{\left(y-y_{N}^{\prime }\right)}^{2}}=r_{N}^{\prime } \end{array}\Leftrightarrow \{\begin{array}{l} {\left(x-x_{1}^{\prime }\right)}^{2}+{\left(y-y_{1}^{\prime }\right)}^{2}=r_{1}^{\prime 2}\\ {\left(x-x_{2}^{\prime }\right)}^{2}+{\left(y-y_{2}^{\prime }\right)}^{2}=r_{2}^{\prime 2}\\ \;\vdots \\ {\left(x-x_{N}^{\prime }\right)}^{2}+{\left(y-y_{N}^{\prime }\right)}^{2}=r_{N}^{\prime 2} \end{array}$$

where (*x, y*) is the location of a sensor node, $\left(x_{i}^{\prime },y_{i}^{\prime }\right)$ is the location of the *i^th^* projected beacon point, *N* is the number of the projected beacon points, and $r_{i}^{\prime }$ is the estimated distance between a sensor node and the *i^th^* projected beacon point.

**Figure 5.**
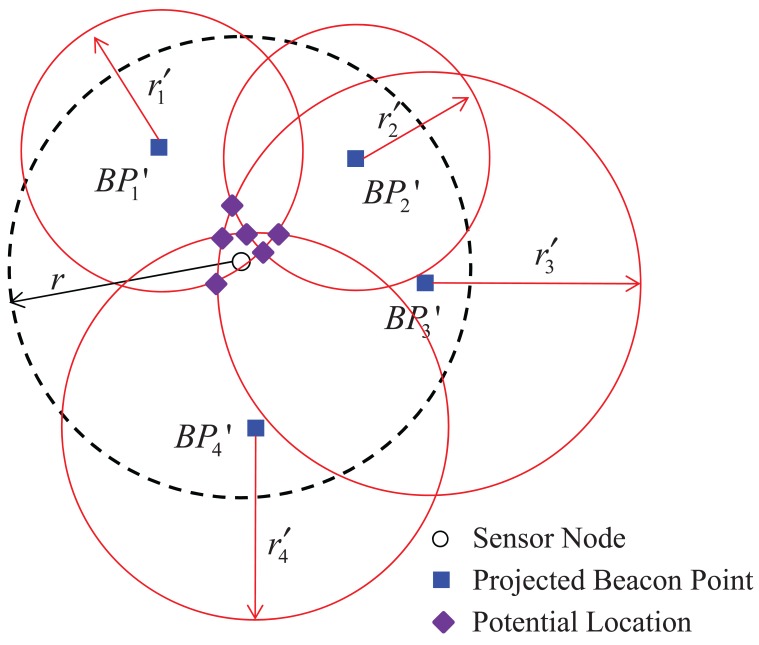
Estimation of the sensor node location using projected beacon points.

**Figure 6.**
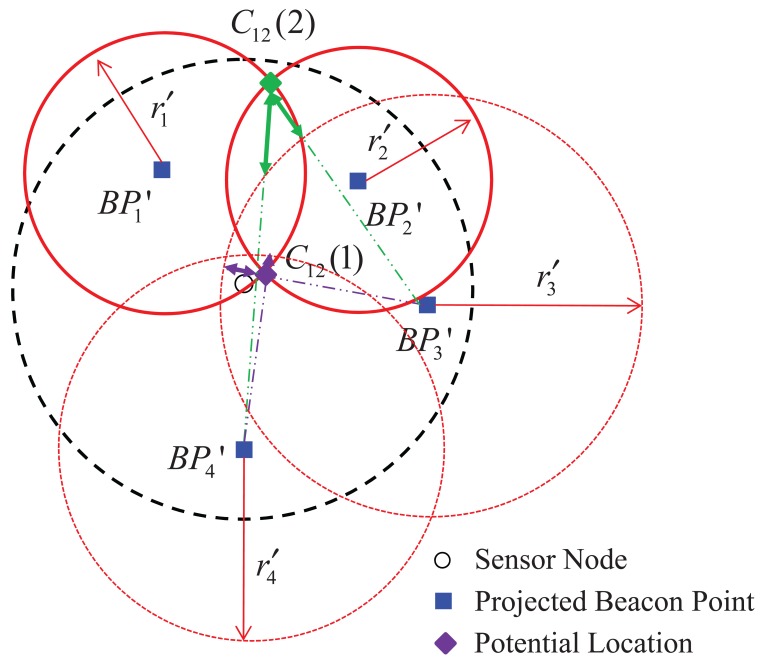
Selection of a potential location between two intersection points.

Let us explain how to estimate the location of a sensor node based on the bilateration method with Figures 5 and 6. When *N* projected beacon points are obtained, *N* circles can be drawn satisfying Equation (2); *i.e.*, four circles with centers $BP_{1}^{\prime }$, $BP_{2}^{\prime }$, $BP_{3}^{\prime }$, and $BP_{4}^{\prime }$ and radii $r_{1}^{\prime }$, $r_{2}^{\prime }$, $r_{3}^{\prime }$, and $r_{4}^{\prime }$ can be drawn, as shown in Figure 5. The points of intersection of the *N* circles that are near the sensor node are obtained as potential locations for the sensor node's estimated location, as shown in Figure 5; some or all of the potential locations may overlap. However, because a potential location can be either of the two intersection points of two circles, a decision process is needed; here, because the intersection points are obtained using just two of many projected beacon points, this method is called bilateration [23]. One of the two intersection points of the two circles with centers at two projected beacon points is closer to the circles with centers at the other projected beacon points than the other intersection point; *i.e., C*_12_(1) is closer to the two circles with centers $BP_{3}^{\prime }$ and $BP_{4}^{\prime }$ than *C*_12_(2) in Figure 6. When *C_jk_*(1) and *C_jk_*(2) are the intersection points of the circles with centers $BP_{j}^{\prime }$ and $BP_{k}^{\prime }$ and the following inequality is satisfied, *C_jk_*(1) is selected as the potential location *P̂_jk_*; otherwise, *C_jk_*(2) is selected.

(3) $$\sum_{i=1,i\neq j,k}^{N}\left|\left\|BP_{i}^{\prime }-C_{jk}(1)\right\|-r_{i}^{\prime }\right|<\sum_{i=1,i\neq j,k}^{N}\left|\left\|BP_{i}^{\prime }-C_{jk}(2)\right\|-r_{i}^{\prime }\right|$$

In rare cases, the number of intersection points between two circles can be one or zero. If there is one intersection point, this single point becomes the potential location. If there is no intersection point, which occurs when the distance between the two beacon points $BP_{j}^{\prime }$ and $BP_{k}^{\prime }$ is larger than $r_{j}^{\prime }+r_{k}^{\prime }$, the point that divides the line segment $BP_{j}^{\prime }BP_{k}^{\prime }$ joining the points $BP_{j}^{\prime }$ and $BP_{k}^{\prime }$ into a $r_{j}^{\prime }:r_{k}^{\prime }$ ratio becomes the potential location. In these two cases, a decision process is not needed.

#### 3.3.2. Estimating the Location of a Sensor Node Using the Weighted Mean

The location of a sensor node can be estimated using all the potential locations. The location of a sensor node can simply be estimated as the mean of all the potential locations. However, to improve the estimation accuracy, a weighted mean of the potential locations can be used instead. Here, the weight is determined based on a residual, which we define here. Given *x̂* as a solution of *x* at *f*(*x*) = *b*, the residual is *b* − *f*(*x̂*), which indicates how far *f*(*x̂*) is from the correct value of *b*, and the error is *x̂* − *x*. As we do not know *x*, we cannot compute the error, but we can compute the residual. From Equation (2) *N* residuals associated with the potential location *P_jk_*(*x̂_jk_, ŷ_jk_*) can be computed as follows:

(4) $$\{\begin{array}{c} {\hat{ɛ}}_{jk}(1)=\left|\sqrt{{\left({\hat{x}}_{jk}-x_{1}^{\prime }\right)}^{2}+{\left({\hat{y}}_{jk}-y_{1}^{\prime }\right)}^{2}}-r_{1}^{\prime }\right|\\ {\hat{ɛ}}_{jk}(2)=\left|\sqrt{{\left({\hat{x}}_{jk}-x_{2}^{\prime }\right)}^{2}+{\left({\hat{y}}_{jk}-y_{2}^{\prime }\right)}^{2}}-r_{2}^{\prime }\right|\\ \vdots \\ {\hat{ɛ}}_{jk}(N)=\left|\sqrt{{\left({\hat{x}}_{jk}-x_{N}^{\prime }\right)}^{2}+{\left({\hat{y}}_{jk}-y_{N}^{\prime }\right)}^{2}}-r_{N}^{\prime }\right| \end{array}$$

Because the accuracy of a potential location is expected to be inversely proportional to the sum of the residuals, the reciprocal of the sum of the residuals represents a weighting factor. The weight for the potential location *P_jk_*(*x̂_jk_, ŷ_jk_*) is defined as follows:

(5) $$w_{jk}=\frac{1}{\sum_{i=1}^{N}{\hat{ɛ}}_{jk}(i)}$$

Finally, the location of a sensor node is estimated as the weighted mean of all the potential locations.

(6) $$(\hat{x},\hat{y})=\frac{\sum_{j=1}^{N-1}\sum_{k=j+1}^{N}w_{jk}({\hat{x}}_{jk},{\hat{y}}_{jk})}{\sum_{j=1}^{N-1}\sum_{k=j+1}^{N}w_{jk}}$$

Less than three beacon points may be acquired for some sensor nodes in certain circumstances. In this case, because the location of a sensor node cannot be estimated through Equation (6), another approach is needed. When just two beacon points $BP_{1}^{\prime }$ and $BP_{2}^{\prime }$ are acquired for a sensor node, the intersection points *C*_12_(1) and *C*_12_(2) of two circles with centers $BP_{1}^{\prime }$ and $BP_{2}^{\prime }$ and radii $r_{1}^{\prime }$ and $r_{2}^{\prime }$ are obtained. Because there are no other beacon points available to the decision-making process, the neighboring sensor nodes can play the role of an additional beacon point in order to estimate the sensor location. The sensor node can receive the location information of neighboring sensor nodes by sending a request to the neighboring sensor nodes that know their location after localization. The sensor node just knows that the neighboring sensor nodes are within the communication range without the distance information between the sensor node and the neighboring sensor nodes. If one of the intersection points is close to the sensor node, more sensor nodes among neighboring sensor nodes of the sensor node are expected to be within the communication range of the intersection point, compared to the other intersection point. By comparing the number of neighboring sensor nodes of the sensor node within the communication range for each intersection point, one of the two intersection points that has more neighboring sensor nodes of the sensor node is estimated as the location of the sensor node. When a sensor node has less than two beacon points, the estimated locations of the neighboring sensor nodes can be used. In this case, the location of the sensor node is estimated as the midpoint of all the neighboring sensor nodes.

It is noteworthy that the main feature of LoMoB that can improve LDB is the use of the weighted mean of all the potential locations. In LDB, when a sensor node has four projected beacon points *F*_1_, *L*_1_, *F*_2_, and *L*_2_, as shown in Figure 3(b), two possible points are obtained based on *F*_1_ and *L*_1_ and one point between the two is estimated as the location of the sensor node based on *F*_2_ and *L*_2_. Here, the error in the estimated location depends largely on the distance between *F*_1_ and *L*_1_ [16]. If *F*_1_ and *L*_1_ are located very close to each other, the location error is expected to be large. In this case, a better choice may be that *F*_2_ and *L*_2_ (or another two projected beacon points) are used for the two possible points, rather than *F*_1_ and *L*_1_. However, the selection of two projected beacon points for the possible points has not yet been studied. If the errors of the beacon points *F*_1_ and *L*_1_ increase because of some factors such as irregularities in the radius of the circle formed by the transceiver's beam and the location error of a mobile beacon, the errors of the two possible points also increase, which results in an increase of the location error. Even though more beacon points, in addition to *F*_1_, *L*_1_, *F*_2_, and *L*_2_, are obtained, the localization accuracy is not improved because the additional beacon points are not used for localization. As in LDB, LoMoB obtains two intersection points based on two beacon points and determines one point between the two as a potential location for the location of the sensor node based on the other beacon points. However, compared to LDB, LoMoB estimates the location of a sensor node based on all the potential locations, not just one potential location. In addition, potential locations with a high weight contribute more to the estimation of the location of the sensor node than other potential locations. For these reasons, LoMoB is expected to improve LDB in challenging underwater environments even though LoMoB uses just four beacon points, as does LDB. If more than four beacon points are used, the localization accuracy is expected to improve further.

## 4. Performance Evaluations

In this section, we first describe the simulation parameters, then introduce the metric to evaluate the localization accuracy and, finally, we compare the localization accuracy of LoMoB with that of LDB through simulations.

### 4.1. Simulation Setup

The sensing space for UASN is a rectangular parallelepiped of 1 km × 1 km × 100 m, in which 100 sensor nodes are randomly deployed. A mobile beacon is assumed to move linearly at a velocity of 1 m/s and broadcasts a beacon at every beacon interval, *i.e.*, each 1 s. To fairly compare LoMoB and LDB, we assume that a mobile beacon has a directional transceiver and just four beacon points are used for localization because LDB works for a system using a directional transceiver and uses four beacon points. The angle of the directional transceiver's beam is 60°. Because it is more convenient to move a mobile beacon on the sea surface than underwater in real underwater environments, the mobile beacon is assumed to move on the sea surface.

In LoMoB and LDB, localization begins by selecting beacon points. Any projected beacon points that are not exactly on the circle formed by the transceiver's beam for a sensor node cause an estimation error of the distance between the projected beacon point and the sensor node, which results in an error in the estimated location. The beacon distance causes projected beacon points to be located not exactly on the circle formed by the beam. In addition, the phenomena such as reflection, diffraction, and refraction in underwater environments cause irregularities in the radius of the circle formed by the beam. To improve the reality of our simulations, irregularities in the radius of the circle are modeled as *r̂* = *r* + *e_r_*, where *e_r_* is a random value with the Gaussian distribution *N* (0, $\sigma_{r}^{2}$). Because the GPS error in a mobile beacon, water fluctuations, and tidal currents can cause errors in the location of the mobile beacon, we assume the location information broadcasted from a mobile beacon is error prone [24]. The location error of a mobile beacon is modeled as **x̂_m_** = **x_m_** + **e_m_**, where **x̂_m_** = [*x̂, ŷ*]^T^ is the measured location of the mobile beacon located at **x_m_** = [*x, y*]^T^ and **e_m_** = [*e_x_, e_y_*]^T^ is a random vector whose *e_x_* and *e_y_* are independent and identical random values with the Gaussian distribution *N*(0, $\sigma_{m}^{2}$). We use Matlab to perform these simulations.

### 4.2. Metrics

To compare the localization accuracy of LoMoB and LDB, we define the average location error as follows:

(7) $$e_{\text{average}}=\frac{1}{N}\sum_{k=1}^{N}\left\|{\hat{x}}_{k}-x_{k}\right\|$$

where **x̂_k_** = (*x̂_k_, ŷ_k_*) is the estimated location of a sensor node that is located at **x_k_** = (*x_k_, y_k_*) and *N* is the number of sensor nodes.

Because the average location error may be seriously affected by a small number of large location errors, the ratio of localized sensor nodes with location errors below a given threshold can be used as an additional metric for evaluating the localization accuracy. The ratio of localized sensor nodes below a given threshold allows the localization accuracy to be assessed in greater depth than the average location error alone.

### 4.3. Simulation Results

Because the beacon distance, irregularities in the radius of the circle formed by the transceiver's beam, and the location error of a mobile beacon all influence the accuracy of distance estimation between a sensor node and a projected beacon point, the localization accuracy is analyzed with respect to these factors.

**Figure 7.**
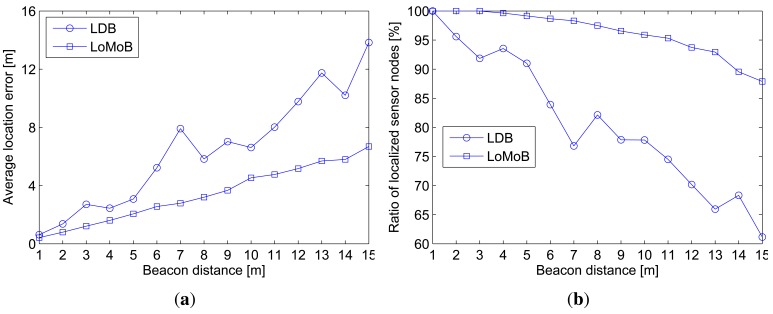
Localization accuracy as a function of beacon distance. (**a**) Average location error; (**b**) Ratio of localized sensor nodes.

Figure 7(a) compares the average location error as a function of the beacon distance and Figure 7(b) compares the ratio of localized sensor nodes with location errors below 10 m. The average location error of LDB is shown to fluctuate more and to be larger than that of LoMoB. As the beacon distance increases, the performance gap between LoMoB and LDB tends to widen. Figure 7 demonstrates that LoMoB is less vulnerable to the beacon distance than LDB.

**Figure 8.**
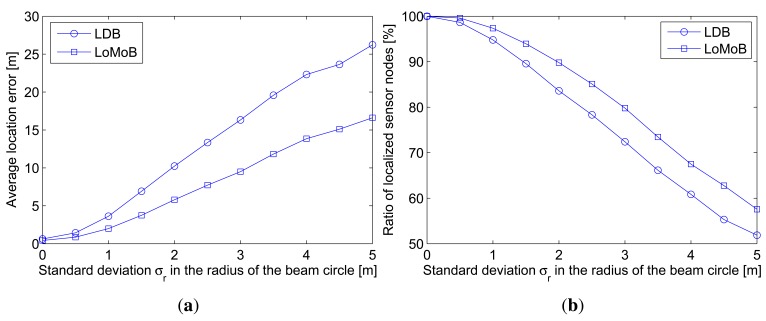
Localization accuracy as a function of standard deviation *σ_r_* in the radius of the circle formed by the transceiver's beam. (**a**) Average location error; (**b**) Ratio of localized sensor nodes.

In real underwater environments, the radius of the circle formed by the transceiver's beam is expected to fluctuate. Irregularities in the radius of the circle cause sensor nodes to provide erroneous estimates of distances between the projected beacon points and a sensor node, in addition to the error associated with the beacon distance. Figure 8(a) compares the average location error as a function of the standard deviation *σ_r_* in the radius of the circle and Figure 8(b) compares the ratio of localized sensor nodes with location errors below 10 m. As shown in the figures, the average location error increases as irregularities in the radius of the circle increase. LoMoB has 32.4%–45.8% smaller location errors than LDB. The ratios of localized sensor nodes show a maximum difference of 7.4%. LoMoB is verified to be more tolerant to irregularities in the radius of the circle formed by the transceiver's beam than LDB.

**Figure 9.**
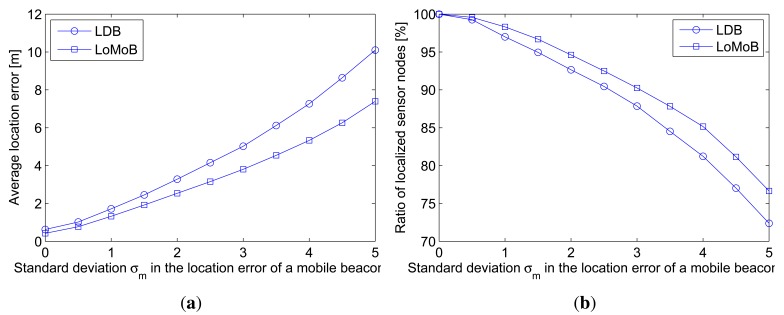
Localization accuracy as a function of standard deviation *σ_m_* in the location error of a mobile beacon. (**a**) Average location error; (**b**) Ratio of localized sensor nodes.

**Figure 10.**
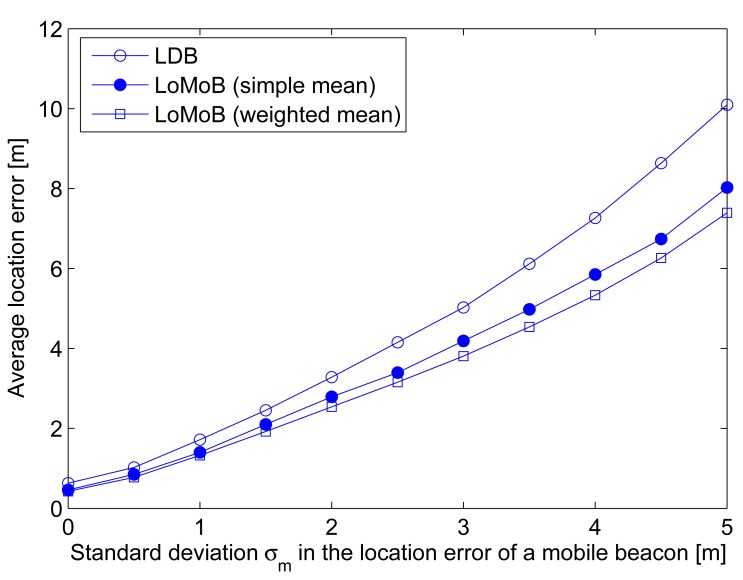
Comparison between simple mean and weighted mean.

A mobile beacon is generally assumed to know its own location. In reality, the location information of a mobile beacon may be erroneous. As well as irregularities in the radius of the circle formed by the transceiver's beam, the location error of a mobile beacon needs to be considered because this also causes erroneous estimation of the distances between the projected beacon points and a sensor node. Figure 9(a) compares the average location error according to the standard deviation *σ_m_* in the location error of a mobile beacon and Figure 9(b) compares the ratio of localized sensor nodes with location errors below 10 m. LoMoB improves the average location error by 21.5%–32.4% compared to LDB. The ratios of localized sensor nodes show a maximum difference of 4.3%. In addition, Figure 10 shows the average location error of LDB and LoMoB applying a simple mean and the weighted mean; if all the weights are equal, then the weighted mean is the same as the simple mean. LoMoB applying a simple mean and the weighted mean improves the average location error by 14.3%–26.9% and 21.5%–32.4%, respectively. This indicates that the use of all the potential locations in LoMoB is the main factor to improve LDB and the use of the weights results in additional increase in the localization accuracy.

The localization accuracy performance of LoMoB and LDB was compared through the average location error and the ratio of localized sensor nodes with location errors below 10 m according to the beacon distance, irregularities in the radius of the circle formed by the transceiver's beam, and the location error of a mobile beacon. The simulation results verify that LoMoB is more tolerant to estimation errors of the distances between the projected beacon points and a sensor node. Subsequently, LoMoB is more promising than LDB in harsh underwater environments.

## 5. Conclusions

In this paper, we proposed a range-free localization scheme for UASN with a mobile beacon that provides the potential locations with weighting factors according to residuals and estimates the location of a sensor node through the weighted mean of the potential locations. Because LoMoB localizes a sensor node based on the weights of the potential locations, it improves the localization accuracy and is more tolerant to errors in the estimation of the distance between projected beacon points and sensor nodes. Simulation results show that LoMoB significantly improves the localization accuracy of LDB, especially in underwater environments that cause irregularities in the radius of the circle formed by the transceiver's beam and the location error of a mobile beacon. Our simulations demonstrated that LoMoB is more robust with respect to errors in distance estimation than LDB.
